# Accuracy of Automatic Carbohydrate, Protein, Fat and Calorie Counting Based on Voice Descriptions of Meals in People with Type 1 Diabetes

**DOI:** 10.3390/nu10040518

**Published:** 2018-04-21

**Authors:** Piotr Ladyzynski, Janusz Krzymien, Piotr Foltynski, Monika Rachuta, Barbara Bonalska

**Affiliations:** 1 Nalecz Institute of Biocybernetics and Biomedical Engineering of the Polish Academy of Sciences, 4 Trojdena Street, 02-109 Warsaw, Poland; piotr.foltynski@ibib.waw.pl; 2 Department of Diabetology and Internal Medicine, Medical University of Warsaw, 1A Banacha Street, 02-097 Warsaw, Poland; janusz.krzymien@hotmail.com (J.K.); m.rachuta@op.pl (M.R.); klindiab@wum.edu.pl (B.B.)

**Keywords:** carbohydrate counting, protein and fat counting, calorie counting, automatic bolus calculator, voice description of meals, insulin dosage, glycemic control, diabetes mellitus

## Abstract

The aim of this work was to assess the accuracy of automatic macronutrient and calorie counting based on voice descriptions of meals provided by people with unstable type 1 diabetes using the developed expert system (VoiceDiab) in comparison with reference counting made by a dietitian, and to evaluate the impact of insulin doses recommended by a physician on glycemic control in the study’s participants. We also compared insulin doses calculated using the algorithm implemented in the VoiceDiab system. Meal descriptions were provided by 30 hospitalized patients (mean hemoglobin A1c of 8.4%, i.e., 68 mmol/mol). In 16 subjects, the physician determined insulin boluses based on the data provided by the system, and in 14 subjects, by data provided by the dietitian. On one hand, differences introduced by patients who subjectively described their meals compared to those introduced by the system that used the average characteristics of food products, although statistically significant, were low enough not to have a significant impact on insulin doses automatically calculated by the system. On the other hand, the glycemic control of patients was comparable regardless of whether the physician was using the system-estimated or the reference content of meals to determine insulin doses.

## 1. Introduction

Technical innovations create many possibilities in supporting the treatment of people with diabetes. According to the Statista Inc. report, smartphone user penetration as a percentage of the total global population exceeded 25% in 2015 [1]. This percentage is forecast to reach 37% by the year 2020. Advances in information and communication technologies (ICT) bring a significant opportunity to develop the integrated healthcare system, which is so difficult to achieve with the current traditional model of healthcare delivery. Telemedicine has the potential to become a key element of future integrated care—an important component of the new healthcare model according to the World Health Organization (WHO) [2]. In 2013, only about 6000 medical applications, i.e., medical “apps”, were available in Google Play for Android-based smartphones [3], and this number increased rapidly in the following years to exceed 45,000 in 2017 [4]. The primary goal of majority of these medical apps is to help and coordinate continuous healthcare at home [5,6]. There is an ongoing discussion as to whether those mobile health (m-health) applications facilitate the gain of clinical benefits, to what extent they can be integrated with the current healthcare system and, finally, whether they are safe and do not create potential health risks for the patient. The US Food and Drug Administration (FDA) classifies the mobile application as a medical device if it is used to prevent, diagnose, care for or cure the disease. Such an app requires the approval of the Agency before it appears on the market [7]. The app should only be recommended to patients by health care professionals if its effectiveness has been scientifically confirmed.

Diabetes is one of the chronic diseases that requires a lot of attention from both the patient and the healthcare team. Regardless of the type of diabetes, patients require full information about the disease through continuous education and promotion of health-seeking behaviors as well as regular glucose monitoring, individual treatment plans, and an early diagnosis to prevent the health threats associated with complications of diabetes. Telemedicine provides a number of tools that could be helpful in choosing the right treatment plan, supporting actions to change a patient’s lifestyle, strengthening motivation regarding health-related activities, facilitating a patient’s ability to self-monitor and control their condition, and achieving the intended therapeutic goal.

Proper dietary treatment is one of the most important components of diabetes therapy, because it significantly affects glycemic control. The growing health awareness of patients has increased the interest in the use of new technologies that can help with dietary intervention and provide nutritional advice. Compared to traditional methods of diet planning and nutrition assessment, new technologies have many advantages, including the ability to quickly provide personalized advice. Several studies have shown that the use of new technologies that provide information and advice on diet can lead to positive changes in the dietary regimen of a patient, affecting the intake of selected nutrients [8]. Although there is still debate regarding the effectiveness of using new technologies in promoting a healthy diet, patients prefer applications that are quickly available and easy to use, increase the awareness of the type of food consumed and facilitating body weight control. In nine randomized controlled trials on the use of smartphone apps for promoting a healthy diet and nutrition, the use of such apps led to the selection of foods recommended by nutritionists, i.e., foods of higher quality, with lower calorific value and low-fat content, as well as participation in significantly more intense physical activity. These changes in the lifestyle resulted in significantly greater weight loss in comparison with people who did not use mobile apps [9]. However, to provide the personalized dietary advice, an appropriate method for measuring and evaluating food intake is required.

The digital revolution has made it possible to develop new instruments for the quantitative assessment of consumed food products [10]. Currently, new solutions supporting the estimation of food consumption use the Internet, mobile technology or both. They are preferred in comparison with traditional methods by both young people and adults. A global consistent increase in Internet access over the last few decades has resulted in the emergence of a number of websites that allow estimation of the consumption of products for both research and commercial purposes [11]. They can be easily accessed using desktop computers, but also from mobile devices, such as tablets or smartphones. In contrast to on-paper nutrition assessment methods, online systems have a few advantages—they can be pre-programmed and digital images of food items can be used to increase food recognition accuracy and facilitate estimation of portion size. There are four basic methods of food coding: the electronic food diary, the photo-assisted tutorial, analysis of food photography by trained dieticians and the automatic analysis of digital food images [12]. Smartphones have enormous potential; apps enable cheap interventions among large populations [13], they make it possible to record data in real time, they are convenient to use, and they can provide continuous monitoring of consumed foods because users usually carry smartphones with them [14].

Automatic or semi-automatic food image analysis systems for dietary assessment are under continuous development. They achieve recognition accuracy below 90% when tested on databases consisting of up to a few hundred images of meals/dishes [15]. In recent years, image transducers have been developed that take serial photos, documenting the consecutive stages of a meal intake and enabling the estimation of the quantity of a leftover, uneaten meal [16]. Some of these lifelogging devices, such as the Microsoft SenseCam camera, along with the data obtained from a conventional food diary, allow for the improvement of the accuracy of calorie intake calculations [17]. Alternative approaches, which are based on the voice description of meals [18,19] or the monitoring of activities related to the meal consumption, e.g., chewing or swallowing [20,21] have been also reported.

Accurate assessment of meals consumed, which includes the correct calculation of carbohydrate exchange units (CU) (and in some applications, also protein and fat) and energy content in a meal, is one of the key elements of type 1 diabetes treatment. It is a challenge for many people with diabetes to estimate the appropriate insulin dose that correctly reflects the size and content of the meal, the pre-prandial glucose level and the expected level of physical activity. This may be one of the reasons why many people with type 1 diabetes do not achieve their therapeutic goals, which is expressed by an elevated level of glycated hemoglobin A1c (HbA1c) of 7.5% (58–64 mmol/mol) or more [22,23]. The prolonged lack of adequate glycemic control in this group of patients results in increased rates of complications and mortality [24]. Difficulties that exist in adjusting the prandial insulin dose are, in many cases, the major cause of both postprandial hypoglycemia and, even more often, hyperglycemia. Therefore, many research works have focused on the evaluation of applications aimed at the improvement of metabolic control, the reduction of the risk of hypoglycemic episodes, body weight reduction and improvement of quality of life as well as decision support regarding prandial insulin dose adjustment based on carbohydrate (CHO) counting. Recently, Tascini et al. pointed out that new insight concerning the effect of dietary macronutrients on postprandial glycemic control confirm that prandial insulin doses should combine CHO counting with protein and fat counting [25]. However, these authors also claimed that a successful application of protein and fat counting requires suitable and usable algorithms to be developed. Therefore, only a few reports so far have calculated prandial insulin doses based on integrated CHO, protein and fat counting with simultaneous evaluation of the accuracy or benefits of these calculations [26,27,28]. None of these reports presented data related to the accuracy of prandial insulin dose calculation based on automatic meal content estimation using a voice description of the meal. Foltynski et al. evaluated the efficacy of such a system (VoiceDiab) in controlling postprandial blood glucose concentrations in persons with type 1 diabetes treated with a continuous, subcutaneous insulin infusion under ambulatory conditions [18]. One of the limitations of that study was the lack of data regarding the meals eaten because patients were treated under ambulatory conditions. Other limitations were the fact that almost 75% of the study group were young patients (<18 years of age) and the mean HbA1c at baseline was 7% (53 mmol/mol), which means that the majority of participants were achieving the recommended target metabolic control according to the American Diabetes Association (ADA). These limitations indicate that it was not possible to determine the difference between the actual meal content and the meal content, which was estimated based on the voice description of the meals provided by the patient. Even if it were possible to calculate such differences, they might have been biased by the fact that participants of that study had, on average, good metabolic control. The accurate estimation of the meal content by the system based on the voice description provided by the person with diabetes is one of the necessary conditions to effectively help such a person calculate the proper insulin dose to compensate for the meal. Such a help is much more desirable in patients who have problems in achieving adequate metabolic control. However, the question arises of whether such patients are able to describe meals verbally in a way that makes it possible to automatically estimate the meal content with an accuracy suitable to calculate insulin doses compensating for these meals. The present study tries to answer this question. This question is also very important from the point of view of the possible application of systems using the voice description of meals in people who do not require exogenous insulin, such as the majority of patients with type 2 diabetes, some women with gestational diabetes and people without diabetes who want to monitor their diet, for example to control their body weight.

The previously published report by Foltynski et al. [18] aimed to evaluate only the effect of the use of the VoiceDiab system on postprandial blood glucose concentrations in ambulatory-treated children and young people with type 1 diabetes. In contrast to that study, the objective of the current work is to assess and demonstrate, for the first time, the accuracy of automatic CHO, protein, fat and calorie counting based on voice descriptions of meals provided by hospitalized adult persons with unstable type 1 diabetes using the VoiceDiab system, in comparison with reference counting results made by the dietitian, and to evaluate the effectiveness of the diabetes treatment, depending on whether the physician determined the insulin dosage based on the composition of meals calculated by the dietitian or whether it was automatically estimated by the VoiceDiab system. We also evaluated the effect of differences between macronutrient counting provided by the system and the dietitian regarding the discrepancy between insulin boluses calculated using the bolus calculator implemented in the voice expert system. Hence, both studies are significantly different not only because of different objectives but also due to their different study groups, the design of each study and the analyzed parameters.

## 2. Materials and Methods

### 2.1. Study Group

During short-term hospitalization because of unstable diabetes, 30 patients with type 1 diabetes treated with continuous, subcutaneous insulin infusion were familiarized with the VoiceDiab expert system and used it to verbally describe meals they intended to eat in order to automatically estimate the amount of CHO, protein, fat and calories in these meals. The inclusion criteria were as follows: 18 to 50 years of age, duration of diabetes ≥1 year and ability to comply with dietary recommendations and hospital procedures. Exclusion criteria included metabolic acidosis, dehydration and electrolytic disorders, other diagnosed endocrine diseases, chronic kidney disease (serum creatinine >1.5 mg/dL), proliferative retinopathy and concomitant infections. The study group consisted of 23 women and 7 men, aged 23.8 ± 4.6 (mean ± SD) years (from 19 to 38 years), with a duration of diabetes of 12.2 ± 6.5 years (from 3 to 26 years) and a varied level of metabolic control, expressed by the mean HbA1c, of 8.4 ± 1.5% (68 ± 16 mmol/mol) (from 6.1 to 12.6%, i.e., from 43 to 114 mmol/mol).

All subjects gave their informed consent for inclusion before they participated in the study. The study was conducted in accordance with the Declaration of Helsinki, and the protocol was approved by the Ethics Committee of the Medical University of Warsaw (KB/16/2014).

For each participant, a unified medical history was collected, concerning diet and eating habits, physical activity, insulin therapy (with particular emphasis on insulin boluses and basal infusion), the number of daily blood glucose tests, the frequency and severity of hypoglycemic episodes, and information about other diagnosed diseases, meditation used, smoking habits and alcohol and drug abuse. The physician analyzed this data to identify factors that could affect the glycemic control of the study participants.

### 2.2. The Voice System Design

The system consisted of an Android-controlled smartphone with the client application communicating wirelessly with servers to perform the following tasks: (1) automatic speech recognition (ASR) and transformation of the voice description of the meal into text; (2) analysis of the textual description to determine the composition of the meal; (3) calculation of the insulin dose compensating the meal according to the algorithm, taking into account either only the CHO content, or the CHO, protein and fat contents in the meal. A detailed description of the system can be found elsewhere [29]. It is noteworthy that the database of the system contains characteristics of 900 unique food products and 5000 terms, facilitating effective speech-to-text conversion, including foods that were present in the hospital menu. However, neither the number of calories, the quantities of CHO, protein and fat that characterize each product, nor any other data stored in the system database, were adapted to the characteristics of the hospital menu.

Safety of the patient is a priority in developing technical systems to support the treatment of people with diabetes. The VoiceDiab system contains a few levels of data input validation and control to ensure that the content of meal is calculated based on real data, i.e., that the system correctly “understands” the verbal description of the meal that the patient intends to deliver: (1) the text that results from the speech-to-text conversion is displayed in full to let the user validate the correctness of the automatic speech recognition; (2) each meal segment is associated with three icons indicating whether the system was able to extract from the voice description of the meal its full characteristics, i.e., the name of the food product, portion size or unit of measure and the number indicating the amount of food (a green icon color indicates that the trait associated with this icon has been recognized on the basis of the verbal description, yellow means that the feature has been recognized using contextual and grammatical analysis of the verbal description and red indicates that the system has not recognized the food segment); (3) for each recognized product, the full characteristics (i.e., name of the product, portion size or unit of measure and the number) are shown together with the estimated total mass and the content of CHO, protein, fat and energy to make it possible for the user to verify the correctness of the data; (4) the user must confirm that the meal was recognized in accordance with the verbal description to activate the bolus calculator; (5) a bolus exceeding the individually-configured threshold triggers the display of a warning message.

### 2.3. Voice System Usage and Built-In Bolus Calculator

Each study participant used the system in the following way. Before starting a meal, the participant verbally described its composition, giving the name and size (either in units such as grams, ounces or liters, or in customary units of measure, such as spoons, cups or portions) of each food product present in the meal. The description was transmitted to the server, and after speech-to-text conversion, each food product was identified and displayed on the smartphone screen for verification by the participant. If the identification failed, a warning message showed that the recognition had been unsuccessful due to an ASR failure or a lack of necessary information in the meal description, e.g., when the patient had specified a food product that was not present in the database of the system. In case of ASR failure, the patient repeated the description of the food product that had not been properly identified. For each recognized product, the system calculated the calorie content and CHO, protein and fat contents in grams.

Upon activation, the bolus calculator summarized the total caloric value, the carbohydrate exchange units (CU) and protein–fat exchange units (PFU) in the whole meal, and finally, the insulin dose required to compensate for the meal. The PFU was calculated using the following equation:

PFU = (4 × Protein [kcal] + 9 × Fat [kcal])/100.(1)

If the PFU is greater than 1.0 a dual-wave bolus is recommended consisting of a simple bolus and a square-wave bolus lasting for 4 to 8 h depending on the value of PFU. The total prandial insulin dose was determined based on the following equation [30]:

IB [U] = CU × ICR + PFU × ICR, where ICR is the insulin to CHO ratio.(2)

The first part of the sum in Equation (2) denotes the amount of insulin administered in the simple bolus and the second part denotes the insulin administered in the square-wave infusion of the variable duration. If the PFU is less than 1.0, then the system reduces it to zero and, consequently, recommends an insulin dose in the form of a simple bolus [30].

Regardless of the data received through the VoiceDiab system, the dietitian carefully calculated the content of each meal based on the weight and exact composition of each food product (provided by a supplier on the product label) present in the meal. The dietitian estimated the caloric value of the meal, CU and PFU, which were treated as the ground true or reference values. Based on the reference values of CU and PFU, the reference insulin doses were calculated manually using the same algorithm that was implemented in the VoiceDiab system. Additionally, a simple bolus (i.e., the first part of the sum in Equation (2) was calculated as if the patient was using a pen insulin injector. For the paired comparison of insulin boluses, the same insulin to CHO ratio (ICR) values were used in the insulin bolus calculations for each patient, which were equal to 1.5 and 1.0 for breakfast and the other meals, respectively. The reference counts of calories, CU, PFU and prandial insulin boluses were used to assess the accuracy and safety of the estimates provided by the VoiceDiab system. We used two ICR values to calculate insulin doses in all patients to account for the most important circadian changes in this parameter, but also to clearly show how differences in the meal contents calculated using both methods were reflected in differences in insulin doses. The VoiceDiab system makes it possible to program values of ICR for 8 time periods with flexible time limits during the day. Thanks to this feature, the circadian rhythm of ICR fluctuates, and changes related to illness or menstruation can also be taken into consideration. The VoiceDiab system cannot automatically estimate ICR. However, there has not been any other automatic bolus calculator reported that could do that. The values of ICR have to be programmed by the physician and they can be altered by the educated patient to adjust for changes in life conditions, e.g., illness.

### 2.4. Impact of the Method of Macronutrient Counting on Glycemic Control

To assess whether the automatic estimation of meal content based on the voice description of a meal can be used to control glycemia, the study group was randomly divided into two subgroups. In the first one, consisting of 14 subjects, aged 23.5 ± 3.8 years with HbA1c equal to 8.6 ± 1.8% (70 ± 20 mmol/mol), the insulin boluses were decided by a physician based on the reference meal content data. In the second one, involving subjects aged 23.7 ± 5.4 years with HbA1c equal to 8.5 ± 1.3% (69 ± 14 mmol/mol), the physician only had access to the data provided by the system when determining the insulin dosage. Each study participant was monitored using the continuous glucose monitoring system.

The following parameters were compared between the subgroups: the mean plasma glucose concentration (PG), the percentage of time when glucose concentration was normoglycemic, i.e., higher than 3.9 mmol/L (70 mg/dL) and lower than 10.0 mmol/L (180 mg/dL) (PNPG), the mean maximum increase in PG after the main meals and the number and duration of hypoglycemic episodes (i.e., glucose concentration equal or lower than 3.9 mmol/L or 70 mg/dL).

### 2.5. Statistical Analysis

The discrepancy in the distribution of the assessed variables from normality was assessed using the Shapiro–Wilk W test. The results indicated that the distribution of the variables differed from the normal distribution. Thus, the non-parametric Wilcoxon signed-rank test was used to analyze the significance of differences between the reference values of caloric content, CU and PFU calculated by the dietitian and the values of these parameters estimated by the VoiceDiab system. The same test was used to analyze differences between prandial boluses calculated according to the above-mentioned algorithm. The statistical analysis was carried out using Statistica version 10 (StatSoft, Inc., Tulsa, OK, USA). All data are presented as means ± SDs and their ranges, i.e., minimum and maximum values. Differences were considered to be statistically significant when *p* < 0.05.

## 3. Results

### 3.1. Accuracy of Macronutrient and Calorie Counting Based on Voice Descriptions of Meals

During their stay in hospital, patients received five meals a day, including three main meals and two snacks. All of the study participants used the VoiceDiab system to verbally describe 535 meals consisting of 1644 food products. The routine hospital diet that was served to participants during the study contained 85 unique food products in different combinations and of different sizes/weights. Plain bread, butter, potatoes, cottage cheese, tomatoes, apples, ham and boiled eggs were repeated in the hospital menu most often. Individual meals consisted of 1 to 6 unique food products. The average breakfast consisted of 4.1 ± 0.5 products, the morning snack, 2.2 ± 0.5 products, lunch, 4.0 ± 0.6 products, the afternoon snack, 1.2 ± 0.6 products, and dinner, 4.1 ± 0.4 products.

Table 1 presents the results of calorie counting done by the dietitian based on accurate, carefully collected data regarding the weights and compositions of meals in comparison with the VoiceDiab system estimates based on approximate information provided by the study participants.

The average calorie content in both snacks estimated by the system did not differ from those calculated by the dietitian. In the case of the main meals, the differences were statistically significant. Overall, the system tended to underestimate the calorie count, but the mean differences were relatively small and equal to −7.2 ± 24.4 kcal (−1.7 ± 6.2%), −55.6 ± 54.8 kcal (−10.8 ± 10.4%) and −6.5 ± 26.0 kcal (−1.2 ± 5.4%) for breakfast, lunch and dinner, respectively.

In the case of each meal, except for lunch, the system estimated values of CU which were higher than those calculated by the dietitian (Table 2). The mean differences were equal to 0.3 ± 0.3 CU (8.8 ± 6.4%), 0.0 ± 0.6 CU (0.6 ± 12.4%) and 0.3 ± 0.2 CU (9.2 ± 5.9%), for the consecutive main meals starting with breakfast. In total, for the three main meals, the difference between the CHO content estimated by the system and by the dietitian was lower or equal to ±1 CU (i.e., ±10 g of CHO) in 96.3% of cases.

The remaining 3.7% estimates differed by not more than ±2 CU. The percentage of the results within the range of ±1 CU was equal to 99.1% for breakfast, 90.8% for lunch and 100% for dinner.

The protein and fat contents were underestimated by the system for the main meals with mean differences of −0.1 ± 0.3 PFU (−3.8 ± 12.5%), −0.5 ± 0.5 PFU (−17.4 ± 17.6%) and −0.1 ± 0.3 PFU (−4.5 ± 9.4%), respectively. The results were not different for the afternoon snack, which only sporadically contained protein or fat. For the morning snack, the mean difference was positive with a large variability between estimates provided by the system and the dietitian (13.3 ± 32.6%).

### 3.2. Effect of Differences in Macronutient Counting on Insulin Doses Estimated Using the Built-In Bolus Calculator of the VoiceDiab System

**Figure 1:**
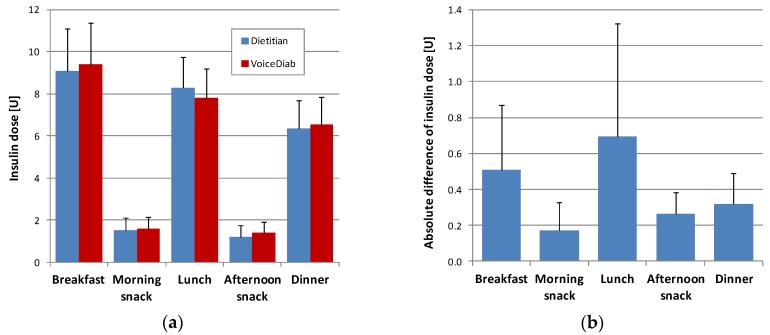
(**a**) Comparison of insulin doses, and (**b**) absolute differences of insulin doses calculated based on carbohydrate (CHO), protein and fat contents provided by the dietitian and the VoiceDiab system.

Figure 1a shows a comparison between the insulin boluses calculated based on meal composition provided by the dietitian versus the system, whereas Figure 1b illustrates the absolute differences between these insulin doses.

In the case of all meals, except for the morning snacks, the differences between insulin boluses were statistically significant (*p* < 0.001). However, the mean absolute difference did not exceed 0.70 U for any meal, and it was below 0.32 U for both snacks and dinner. The mean daily prandial insulin dose in all full days of hospitalization was equal to 25.6 ± 4.6 U when calculated based on the meal content estimated by the dietitian and 25.8 ± 4.4 U when the estimates provided by the VoiceDiab system were used, meaning that the average difference was equal to just 0.2 ± 0.8 U (*p* = 0.059).

**Figure 2:**
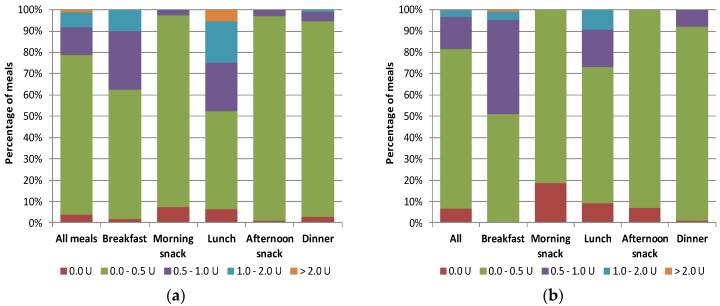
Mean relative absolute differences of insulin doses calculated according to Equation (2) based on estimates of carbohydrate exchange units (CU) and protein–fat exchange units (PFU) provided by the dietitian and the VoiceDiab system for (**a**) dual-wave boluses; (**b**) simple boluses. The insulin to carbohydrate ratio (ICR) was assumed to be 1.5 U/CU for breakfast and 1.0 U/CU for all other meals.

Figure 2a shows, for each meal and for all meals together, the percentage of the prandial insulin doses calculated based on the meal estimates made by the system which were equal to their reference values, those that were in the range of 0.0–0.5 U, 0.5–1.0 U, 1.0–2.0 U and those that differed by more than 2 U from the reference values. The majority of the insulin doses (78.7%) differed by ± 0.5 U at most from the reference values and only 1.3% went beyond the ±2 U range. When we used the values of CU to calculate simple insulin boluses (Figure 2b), neglecting the protein and fat contents in meals, the results were similar, i.e., 81.7% of boluses were different from their reference values by 0.5 U or less, and only 1.1% differed by more than 2 U (of which 0.9% concerned insulin doses compensating breakfast).

### 3.3. Impact of the Method of Macronutrient Counting on Glycemic Control

The average PG and PNPG were similar for both subgroups, i.e., 7.3 ± 0.8 mmol/L (131 ± 15 mg/dL) vs. 7.5 ± 0.9 mmol/L (135 ± 16 mg/dL), and 76 ± 7% (*p* = 0.42) vs. 75 ± 7% (*p* = 0.79), respectively. The maximum increase in PG was equal to 4.3 ± 1.4 mmol/L (77 ± 25 mg/dL) vs. 4.7 ± 1.8 mmol/L (85 ± 33 mg/dL) (*p* = 0.37) after breakfast, 3.7 ± 1.7 mmol/L (67 ± 30 mg/dL) vs. 4.0 ± 1.7 mmol/L (72 ± 30 mg/dL) (*p* = 0.55) after lunch and 3.9 ± 1.3 mmol/L (71 ± 24 mg/dL) vs. 4.3 ± 0.9 mmol/L (77 ± 17 mg/dL) (*p* = 0.11) after dinner, in the first and the second subgroups, respectively. In the first subgroup, hypoglycemia episodes occurred 2.1 ± 0.8 times per day, whereas in the second subgroup, they occurred 2.0 ± 1.3 per day (*p* = 0.77). The daily duration of hypoglycemic episodes was equal to 120 ± 70 min in the first subgroup and 95 ± 74 min in the second subgroup (*p* = 0.35).

## 4. Discussion and Conclusions

Since the mid 1990s, ICT technology has been used to support the treatment of people with type 1 diabetes, based primarily on the telemonitoring of patients’ metabolic states and courses of treatment as well as teleconsultations. Telehome care systems have been used to support type 1 diabetes treatment in a few clinical trials, demonstrating a few benefits of this type of the care over routine periodical check-ups of patients’ states in the physician’s office [31,32,33,34,35]. Rapid development and a widespread use of smartphones created the basis for the development and clinical validation of m-health solutions, making it possible to monitor or support the treatment of people with diabetes in real time [36,37]. Currently, using smartphones, it is possible to transfer a lot of data to the treatment team, such as the results of glucose monitoring, meal size and composition or information on physical activity. However, the more data that is transferred, the more time and effort the therapeutic team needs to analyze these data and effectively support patients. Automatic bolus calculators can reduce the burden on the treatment team, helping patients to adjust their insulin doses to the size and composition of meals [38]. As indicated in the study by Franc et al., frequent support of treatment with a smartphone coupled to a website and the use of automatic bolus calculators may lead to a significant reduction in HbA1c in people with poorly-controlled type 1 diabetes. With less frequent use of smartphone apps, it is beneficial to frequently use teleconsultation services [39]. However, determination of the appropriate dose of insulin administered before the meal depends on the relatively accurate assessment of a meal by calculating, primarily, its CHO content.

The ability of the patient to accurately estimate the size of a meal depends on many factors, including quality of education, frequency of recurrent training and daily practice of such calculations by the patient. According to the survey carried out in people with type 1 diabetes and poor glycemic control, the average error in CHO counting in meals consumed during the day (three main meals and two snacks) was equal to 4.2 CU, and it tended to increase in people with long-term diabetes subjected to systematic education [40]. Brazeau et al., analyzed the differences between CHO estimates made by patients with long-term type 1 diabetes and calculations carried out by dieticians using a computer analysis program and found that the average absolute difference was equal to 15.4 ± 7.8 g (20.9 ± 9.7%) of CHO per meal, which, on average, contained 72.4 ± 34.7 g of CHO [41]. In this study, the CHO content in 63% out of the total of 448 meals was underestimated. Bishop et al. showed, by analyzing the most frequently eaten foods, that, in a group of teenagers, only 23% were able to estimate CHO content with an error not exceeding ±10 g in their daily diet despite the selection of common meals [42]. In up to 52% of these teenage patients, the difference between their calculations and those made by dieticians was within the range of ±30 g. Similar mistakes in CHO counting are made by adults with type 1 diabetes [43]. Currently, new systems are emerging which are aimed at supporting people with type 1 diabetes in CHO counting. The GoCARB system uses computer vision technology for this purpose. The user places a reference card next to the meal and takes two images using a smartphone camera. The system was developed based on the following minimum assumptions: the image contains only one dish/plate, which must be round, and various food products are not mixed on the plate. After taking photos, the images are transmitted to a dedicated server via a WiFi network, where a series of computer image processing operations are performed. All computer vision modules operate on the server, while the mobile phone is used only to acquire images, calculate CU and visualize the results. A comparison was made between the calculations performed by people with type 1 diabetes without system support and with the use of the system. An error below ±20 g per meal from the total of 114 meals was noted in 58.8% and 80.7% of participants, respectively, showing the advantage of using the system [44]. The caloric, CHO, protein and fat contents in food products can be calculated using numerous available publications as well as computer programs and mobile apps that have built-in calculators or databases to facilitate the capacity to obtain information on foods [45]. Often the quality of calculations is affected by the size of the portions.

Patients with type 1 diabetes often face a difficult choice of whether they need only to account for the CHO content of the meal or whether they should also include the contents of protein and fat to determine the insulin dose. Bell et al. attempted to determine differences between postprandial glucose concentrations after eating a high-fat, high-protein meal compared to a low-fat and low-protein meal with the same CHO content, and to determine differences in the insulin doses that should be applied following each of these meals to achieve the best postprandial glycemic control. The authors showed that in the case of a meal containing 40 g of fat and 27 g of protein in addition to 50 g of CHO, the insulin dose should be increased by 65 ± 10% using a dual-wave bolus carried out for 2.4 h to achieve adequate post-meal glycemic control [46]. Wolpert et al. assessed that the consumption of a high-fat lunch caused an average increase in insulin demand of about 42% with significant differences in individual patients [47]. A few studies have shown that the consumption of high-protein and high-fat meals results in an increase and delay in the postprandial glucose rise [48,49], indicating the need to include these nutrients in the determination of insulin doses. The VoiceDiab system make it possible to compensate for such meals using the dual-wave insulin bolus. Nevertheless, it is also possible to pre-program the system to calculate insulin doses based solely on CHO content in meals to be administered in a form of a simple bolus or an injection with a pen injector.

The question remains as to what extent errors in the calculation of CU affect glycemic control. Based on studies in groups of children and adolescents with diabetes, it has been demonstrated that an inaccuracy of ±10 g does not impair postprandial glycemia [49], but a discrepancy of ±20 g or more significantly influences glycemic control after the meal [50]. In spite of the fact that in our study, differences between CU counts estimated by the VoiceDiab system and the dietitian were significant from a statistical point of view, they were lower than ±10 g of CHO in more than 96% of the analyzed main meals and in 100% of snack meals. They were lower than ±20 g in the remaining 4% of the main meals. This result shows that inaccuracies that are introduced both by patients during subjective estimation of the size of a meal, and by the system which uses average characteristics of food products, should not have a significant negative impact on the setting of insulin doses and consequently, on postprandial glycemic control. Such a statement is further confirmed by (1) the results of the application of the VoiceDiab system in a group of ambulatory patients with type 1 diabetes characterized by good metabolic control and treated using insulin pumps, in whom the system proved to be effective in increasing the percentage of 2-hour postprandial glucose in the normoglycemic range by applying the insulin bolus algorithm implemented in the VoiceDiab system [18]; and (2) the results of the glycemic control of the participants of this study, where an expert determined the insulin dosages.

Through analyzing and summarizing the impact of differences in CU and PFU calculations on the resultant insulin doses estimated according to the algorithm implemented in the VoiceDiab system, we demonstrated that in over 91% of meals, the absolute difference in insulin doses were smaller than 1 U. The biggest discrepancies were noted in the calculation of pre-lunch insulin doses, where 26% of the differences were larger than 1 U.

Confirmation of the ability of the VoiceDiab system to provide meal content estimates that are similar to those calculated by a dietitian is an important step on the way to the further utilization of modern technology to support people with type 1 diabetes as well as other groups of patients who need to estimate the macronutrient contents of their meals. However, from the type 1 diabetes perspective, it should be emphasized that the automatic bolus calculator is just one element of the whole infrastructure that should be present to make it possible to adequately train and follow-up people with diabetes and ensure safe and efficient usage of new technology. This infrastructure is necessary for the patients but also for clinicians who have expertise in insulin management and are willing to accept the responsibility for ensuring that each patient receives adequate training and follow-up [51]. To reduce the risk of patients using inappropriate parameters in their daily regimens, patients should fully understand how to use bolus calculators. Otherwise, they may be exposed to the avoidable risk of potentially dangerous changes in glycemic control. Hirsch and Parkin, in their report on the safety and efficacy of smartphone bolus calculator apps, listed four key components of automated bolus calculator training: (1) determine the patient’s competency in utilizing insulin therapy and self-management skills; (2) assess the appropriateness of the patient’s basal dose and key insulin parameters, including the insulin sensitivity factor and ICR, blood glucose targets, and prescribed dosage adjustments for exercise and changes in health status; (3) utilize structured self-monitoring of blood glucose with patients; (4) monitor patient therapy consistently [51]. Generally, these should be key components of the training of any person with type 1 diabetes regardless of whether she or he is going to use an automatic bolus calculator. In fact, all these components were a part of the treatment plan or the education program of the patients participating in the current study. However, due to the study’s main purpose and the fact that after hospitalization, the study participants had not been using the VoiceDiab system, it was not determined whether it would be possible to reproduce the results that were obtained in younger patients with good metabolic control in the previously reported study in this study group of individuals under ambulatory conditions [18]. Nevertheless, during hospitalization, the current study participants achieved lower mean PG values than those participating in the other study.

The meal content data estimated by the system can also be effectively used by the physician in a less formally-defined expert algorithm to effectively manage the intensive insulin treatment; this was confirmed by the comparable glycemic control of study participants whose insulin boluses were determined by the physician based on the reference meal data and those for whom meal content data calculated by the system was used. The differences were not significant regardless whether we analyzed indices characterizing the average daily glycemia, the glucose concentration rise after meals or the frequency and duration of hypoglycemic episodes. However, the lack of differences in glycemic control between both subgroups during this study should be interpreted with due caution, because, despite the fact that efforts were made to exclude patients with diagnosed endocrine disorders other than diabetes, it cannot be ruled out that participants might have had other medical conditions affecting their glycemic control. It should be mentioned, however, that none of the patients reported any coexistent disease or use of medications that may affect glycemic control. Hence, based on the results of the medical history that was collected, it can be stated that in case of the study participants, the most probable causes of unstable metabolic control were related to diabetes and included inappropriate health behaviors, insufficient daily blood glucose tests, fear of hypoglycemia and difficulties related to the proper adjustment of the bolus to the meal content. Nevertheless, participants could have intentionally or accidentally not informed the physician about medical conditions affecting their glycemic control.

Summing up, people with type 1 diabetes, despite education, face several difficulties in adjusting their insulin dosage based on their own estimates of the CHO content of meals. These difficulties may be even more pronounced when a complex insulin bolus is determined to compensate for not only CHO, but also protein and fat content. The developed system, which uses an intuitive user interface, is simple to use and quickly provides information on meal composition that may be used to automatically calculate prandial insulin doses. The obtained results and the literature data indicate that the accuracy of CU and PFU estimates computed by the system is sufficient to calculate insulin doses, either automatically using the algorithm implemented in the VoiceDiab system, or manually using the algorithm based on the knowledge and experience of a physician; these doses were also shown to be close to those calculated based on the reference values of CU and PFU established by the dietitian.

## Figures and Tables

**Table 1:** Calorie content estimated by the dietician and the VoiceDiab system.

Meal	*N*	Calorie Content (kcal)		
		Dietician Mean ± SD ^1^ Min–Max	System Mean ± SD Min–Max	*p*
Breakfast	110	388 ± 85 166–602	381 ± 84 159–586	<0.0001
Morning snack	80	92 ± 38 23–324	90 ± 28 25–238	0.43
Lunch	130	507 ± 98 338–806	451 ± 97 283–766	<0.0001
Afternoon snack	101	58 ± 31 46–163	58 ± 28 34–168	0.46
Supper	114	410 ± 86 221–661	403 ± 79 217–661	<0.0001

^1^ SD, the standard deviation.

**Table 2:** Carbohydrate exchange unit (CU) and the protein–fat exchange unit (PFU) counting by the dietician and the VoiceDiab system.

Meal	*N*	Carbohydrate Exchange Units (CU)			Protein-Fat Exchange Units (PFU)		
		Dietician Mean ± SD Min–Max	System Mean ± SD Min–Max	*p*	Dietician Mean ± SD Min–Max	System Mean ± SD Min–Max	*p*
Breakfast	110	3.8 ± 0.8 2.2–6.0	4.1 ± 0.9 2.4–6.5	<0.0001	2.3 ± 0.6 0.7–3.7	2.2 ± 0.6 0.6–3.4	<0.0001
Morning snack	81	1.1 ± 0.4 0.5–3.1	1.1 ± 0.5 0.5–3.2	0.01	0.4 ± 0.3 0.0–1.9	0.4 ± 0.2 0.0–1.1	0.01
Lunch	130	5.5 ± 1.0 2.3–9.1	5.5 ± 1.0 2.9–8.0	0.78	2.8 ± 0.8 1.2–5.1	2.3 ± 0.8 0.7–5.2	<0.0001
Afternoon snack	100	1.2 ± 0.4 1.0–2.6	1.3 ± 0.4 0.9–2.5	<0.0001	0.05 ± 0.14 0.0–0.5	0.05 ± 0.13 0.0–0.6	0.28
Supper	114	3.9 ± 0.9 2.3–6.7	4.2 ± 0.9 2.7–6.5	<0.0001	2.5 ± 0.6 0.8–4.4	2.3 ± 0.5 0.7–4.2	<0.0001

## Supplementary Materials

The following are available online at http://www.mdpi.com/2072-6643/10/4/518/s1, Table S1: Basic characteristics of the study group, Table S2: Carbohydrate exchange units (CU), protein–fat exchange units (PFU) and calorie contents of meals calculated by the dietitian and estimated by the VoiceDiab system.
