# Supplementary material for: Accuracy of Automatic Carbohydrate, Protein, Fat and Calorie Counting Based on Voice Descriptions of Meals in People with Type 1 Diabetes
Source: Nutrients. 2018 Apr 21;10(4):518. doi: 10.3390/nu10040518 (PMC5946303; doi:10.3390/nu10040518)
Supplement: Supplementary file 1 [file nutrients-10-00518-s001.pdf]

Table S1. Basic characteristics of the study group.

Pat. no.	Age [years]	Duration of diabetes [years]	Hemoglobin A1c [%]	Hemoglobin A1c [mmol/mol]
1	22	6	10.0	86
2	19	7	6.1	43
3	27	16	8.3	67
4	20	11	8.6	70
5	19	13	7.6	60
6	22	8	8.9	74
7	28	25	8.3	67
8	24	5	7.6	60
9	20	7	8.0	64
10	27	20	7.7	61
11	19	16	9.7	83
12	21	11	8.7	72
13	21	8	8.3	67
14	29	13	6.4	46
15	19	5	8.4	68
16	28	20	8.0	64
17	38	3	7.7	61
18	20	10	7.7	61
19	28	3	12.6	114
20	21	13	10.6	92
21	25	15	6.8	51
22	24	11	10.1	87
23	19	3	6.8	51
24	19	13	11.0	97
25	25	6	8.2	66
26	30	23	10.1	87
27	22	10	6.4	46
28	31	25	8.1	65
29	25	17	6.4	46
30	22	11	9.2	77

Table S2. Carbohydrate exchange units (CU), protein-fat exchange units (PFU) and calorie content (kcal) of meals calculated by the dietitian and estimated by the VoiceDiab system.

Pat. no.	Day	Meal [1]	Dietitian			VoiceDiab		
			CU	PFU	kcal	CU	PFU	kcal
1	1	1	5.5	2.2	460	5.0	3.2	521
1	1	3	5.3	3.0	528	5.1	2.3	433
1	1	4	1.0	0.0	70	1.3	0.0	51
1	1	5	3.2	2.3	363	3.5	2.6	406
1	2	1	3.0	1.5	290	3.5	1.8	314
1	2	2	1.0	0.4	77	1.3	0.4	92
1	2	3	6.0	3.0	534	6.6	2.5	508
1	2	4	1.0	0.0	70	1.3	0.4	92
1	2	5	3.4	1.5	297	2.7	1.8	285
1	3	1	3.3	2.0	335	3.5	2.3	370
1	3	2	0.8	0.6	102	1.2	0.6	103
1	3	3	4.7	2.4	481	4.2	2.6	427
1	3	4	1.0	0.0	46	1.3	0.0	51
1	3	5	3.7	1.9	349	4.2	1.8	346
1	4	1	3.5	1.5	343	3.9	1.8	332
1	4	2	0.6	0.3	56	1.0	0.4	78
1	4	3	5.9	2.8	590	6.6	2.1	475
1	4	4	1.0	0.0	46	1.3	0.0	51
1	4	5	3.5	2.0	350	3.9	2.1	372
1	5	1	3.6	1.1	342	4.0	1.5	309
1	5	2	0.8	0.3	72	1.1	0.4	85
1	5	3	5.0	3.6	570	5.4	1.6	376
2	1	1	5.0	2.0	420	3.3	2.4	374
2	1	2	1.5	0.3	100	1.1	0.4	85
2	1	3	6.7	2.7	600	7.0	1.9	471
2	1	4	1.0	0.0	50	1.3	0.0	51
2	1	5	5.8	3.0	540	6.1	2.4	483
2	2	1	5.3	2.4	459	4.6	2.3	419
2	2	2	1.2	0.5	99	1.3	0.4	92
2	2	3	7.2	3.9	695	6.7	3.0	567
2	2	4	2.0	0.4	131	2.5	0.6	156
2	2	5	6.3	3.5	616	5.8	3.5	582
2	3	1	5.8	3.0	540	5.3	2.5	456
2	3	2	2.0	0.4	131	2.5	0.4	136
2	3	3	8.0	4.4	768	6.9	3.1	584
2	3	4	2.0	0.4	131	2.5	0.4	136
2	3	5	5.4	3.3	552	5.8	2.0	432
2	4	1	5.2	3.7	587	5.7	3.2	552
2	4	2	2.2	0.4	138	2.6	0.4	143
2	4	3	6.1	3.2	567	7.1	2.1	492
2	4	4	2.1	0.4	126	2.5	0.4	136
2	4	5	5.6	3.3	564	6.2	2.4	490
2	5	1	5.4	2.8	500	5.8	2.8	506
2	5	2	2.6	1.1	222	2.6	0.9	192
2	5	3	9.1	4.2	800	8.0	3.2	640
3	1	1	3.2	0.9	229	3.9	1.2	276
3	1	2	1.0	0.4	85	0.9	0.6	93
3	1	3	5.2	2.3	449	4.0	2.6	420
3	1	4	1.0	0.0	50	1.3	0.0	51
3	1	5	3.4	1.9	333	3.9	1.9	349
3	2	1	3.3	2.3	349	3.4	1.9	330

Pat. no.	Day	Meal [1]	Dietitian			VoiceDiab		
			CU	PFU	kcal	CU	PFU	kcal
3	2	2	0.9	0.4	77	1.0	0.4	78
3	2	3	5.1	3.3	546	4.9	2.3	421
3	2	4	1.0	0.0	46	1.3	0.0	51
3	2	5	3.5	2.3	360	3.9	1.9	348
3	3	1	3.6	2.3	359	3.9	1.9	346
3	3	3	6.7	2.9	592	6.9	2.4	516
3	3	4	1.0	0.0	50	1.3	0.0	51
3	3	5	3.2	1.9	326	3.5	2.3	376
3	4	1	3.3	1.9	326	3.5	2.3	370
3	4	2	1.0	0.7	114	1.2	0.6	103
3	4	3	5.0	2.0	412	4.8	1.8	363
4	1	1	3.5	2.2	372	4.0	2.2	376
4	1	2	1.1	0.3	80	1.2	0.3	75
4	1	3	4.4	2.4	419	4.6	1.9	374
4	1	5	3.2	1.9	326	3.5	1.7	309
4	2	1	3.2	1.9	326	3.5	1.7	309
4	2	3	6.6	1.9	461	6.6	1.9	446
4	2	5	4.6	2.0	390	3.9	2.8	432
4	3	1	3.5	2.2	372	4.0	2.2	376
4	3	3	5.8	3.0	543	5.8	2.5	482
4	3	5	3.4	2.5	392	3.6	1.8	370
4	4	1	3.3	2.8	414	3.4	1.9	383
4	4	3	6.0	2.8	530	6.5	1.7	426
4	4	5	3.2	3.5	485	3.5	2.8	480
5	1	3	6.5	3.9	667	5.5	2.8	488
5	1	4	2.6	0.4	150	2.5	0.3	126
5	1	5	5.8	3.0	540	6.3	2.2	478
5	2	1	3.4	2.5	399	3.9	2.4	392
5	2	2	1.3	0.7	125	1.3	0.4	92
5	2	3	5.8	4.1	654	5.2	2.8	489
5	2	4	2.5	0.5	163	2.5	0.3	126
5	2	5	5.3	3.3	545	5.7	2.4	467
5	3	1	3.3	2.5	395	3.5	1.9	334
5	3	2	1.8	0.7	152	1.8	0.6	131
5	3	3	2.3	2.7	363	4.3	2.0	370
5	3	4	2.5	0.5	162	2.5	0.5	168
5	3	5	5.8	3.0	542	6.4	2.5	505
5	4	1	3.3	3.3	469	3.9	2.8	434
5	4	2	1.3	0.7	124	1.3	0.4	92
5	4	3	5.2	2.9	503	5.5	2.5	462
5	4	4	2.5	0.5	162	2.0	0.3	112
5	4	5	5.4	3.3	550	5.8	2.4	468
5	5	1	3.7	2.9	444	4.1	1.8	342
5	5	2	1.8	0.7	119	1.7	0.3	103
5	5	3	4.0	4.5	501	3.9	2.0	358
6	1	2	1.7	0.4	111	1.6	0.3	93
6	1	3	7.3	3.1	453	6.6	1.2	373
6	1	4	2.1	0.4	125	2.1	0.3	114
6	1	5	5.5	2.8	617	5.7	2.8	513
6	2	1	5.3	3.1	527	5.7	2.9	520
6	2	3	5.9	3.2	568	6.9	3.0	580
6	2	5	5.6	2.8	508	6.0	2.6	505
6	3	1	5.3	3.3	544	5.7	3.1	538
6	3	2	1.7	0.6	135	1.8	0.6	132

Pat. no.	Day	Meal [1]	Dietitian			VoiceDiab		
			CU	PFU	kcal	CU	PFU	kcal
6	3	3	7.3	4.1	715	7.7	3.8	690
6	3	5	6.7	3.4	612	6.5	3.6	618
6	4	1	5.7	3.5	581	6.2	3.4	586
6	4	2	1.3	0.6	119	1.5	0.6	119
6	4	3	6.5	3.8	644	6.4	3.7	616
6	4	5	5.6	3.3	557	6.0	3.1	552
6	5	1	6.0	3.6	602	6.2	3.2	572
6	5	3	6.0	3.6	544	6.4	3.1	564
7	1	1	3.4	2.2	365	3.7	2.2	364
7	1	3	4.1	2.4	411	4.5	2.3	411
7	1	4	1.0	0.0	46	1.1	0.0	41
7	1	5	3.2	1.9	326	3.5	1.8	319
7	2	1	3.2	2.1	350	3.5	2.0	343
7	2	2	1.1	0.3	82	1.0	0.4	78
7	2	3	5.0	2.2	423	3.9	1.5	307
7	2	4	1.0	0.0	46	1.3	0.0	51
7	2	5	3.4	1.9	334	3.9	1.8	333
7	3	1	3.2	1.9	326	3.5	1.8	319
7	3	3	4.8	2.6	459	5.3	1.9	399
8	1	3	5.2	2.3	441	5.3	2.3	442
8	1	4	1.0	0.0	46	0.9	0.0	34
8	1	5	3.7	2.6	406	4.1	2.4	405
8	2	1	3.2	1.0	250	3.4	1.0	233
8	2	2	1.1	0.3	82	1.0	0.4	78
8	2	3	5.2	2.5	462	4.8	2.1	404
8	2	4	1.0	0.0	46	1.3	0.0	51
8	2	5	3.3	2.2	359	3.8	2.1	358
8	3	1	3.7	2.5	406	4.1	2.4	405
8	3	2	1.1	0.3	79	1.2	0.3	80
8	3	3	5.1	3.1	406	5.2	2.9	497
8	3	4	1.0	0.0	46	1.3	0.0	51
8	3	5	3.4	0.8	221	3.7	0.7	217
8	4	1	3.3	1.2	261	3.5	0.7	208
8	4	2	1.1	0.3	82	1.0	0.4	78
8	4	3	5.6	1.9	421	5.0	1.3	323
8	4	4	1.0	0.0	46	1.3	0.0	51
8	4	5	3.3	2.1	351	3.8	2.0	351
8	5	1	3.2	1.3	268	3.5	1.4	281
8	5	2	1.1	0.7	119	1.1	0.7	112
8	5	3	4.9	3.0	500	5.1	2.4	442
9	1	3	5.2	1.2	338	5.3	0.7	285
9	1	4	1.0	0.0	46	1.3	0.0	51
9	1	5	3.7	2.6	406	4.1	2.4	405
9	2	1	3.5	2.5	396	3.8	2.4	391
9	2	2	1.1	0.3	82	1.0	0.4	78
9	2	3	5.9	3.0	540	6.2	1.6	412
9	2	4	1.0	0.0	46	1.3	0.0	51
9	2	5	3.4	2.8	422	3.8	2.7	421
9	3	1	3.7	2.5	406	4.1	2.4	405
9	3	2	1.0	0.7	112	0.9	0.7	108
9	3	3	5.0	4.2	621	4.9	3.2	522
9	3	5	3.3	3.2	460	3.6	3.1	457
9	4	1	3.2	2.4	374	3.4	2.4	371
9	4	3	5.9	1.2	350	5.5	0.7	288

Pat. no.	Day	Meal [1]	Dietitian			VoiceDiab		
			CU	PFU	kcal	CU	PFU	kcal
9	4	4	1.0	0.0	46	1.3	0.0	51
9	4	5	3.3	3.2	462	3.8	3.1	462
9	5	1	3.2	2.4	374	3.5	2.3	367
9	5	3	5.9	1.5	393	6.1	1.5	394
10	1	3	5.7	2.6	494	4.9	2.4	437
10	1	4	1.0	0.0	46	1.3	0.0	51
10	1	5	3.2	1.9	326	3.5	1.8	319
10	2	1	3.2	1.9	326	3.5	1.8	319
10	2	2	1.1	0.3	79	1.1	0.7	117
10	2	3	5.3	2.6	479	4.3	2.3	405
10	2	4	1.0	0.0	46	1.3	0.0	51
10	2	5	3.7	2.5	406	4.1	2.4	405
10	3	1	3.4	1.9	334	3.9	1.8	333
10	3	2	0.8	0.5	91	0.8	0.6	92
10	3	3	6.3	2.8	541	5.2	2.8	543
10	3	4	1.0	0.0	46	0.9	0.0	34
10	3	5	3.5	2.5	398	3.8	2.4	391
10	4	1	3.2	1.9	326	3.5	1.8	319
10	4	2	1.1	0.3	82	1.0	0.4	78
10	4	3	4.7	2.8	476	5.0	1.6	356
10	4	4	1.0	0.0	46	0.9	0.0	34
10	4	5	3.3	3.2	462	3.8	3.1	462
10	5	1	3.7	2.5	406	4.1	2.4	405
10	5	2	0.8	0.4	79	0.9	0.5	80
10	5	3	5.1	3.3	541	5.2	3.0	506
10	5	4	1.0	0.0	46	1.3	0.0	51
10	5	5	3.2	1.9	326	3.5	1.8	319
11	1	3	5.7	2.6	494	5.2	2.5	463
11	1	4	1.0	0.0	46	1.3	0.0	51
11	1	5	3.2	1.9	326	3.5	1.8	319
11	2	1	3.2	1.9	326	3.5	1.8	319
11	2	2	1.1	0.3	79	1.1	0.4	76
11	2	3	5.5	2.7	482	4.5	1.9	373
11	2	4	1.0	0.0	46	1.3	0.0	51
11	2	5	3.7	2.5	406	3.7	2.5	395
11	3	1	3.4	1.9	334	3.9	1.8	333
11	3	2	0.8	0.5	91	0.9	0.6	91
11	3	3	6.1	2.2	475	6.3	2.2	476
11	3	4	1.0	0.0	46	0.9	0.0	34
11	3	5	3.5	2.5	398	3.8	2.4	391
11	4	1	3.2	1.9	326	3.5	1.8	319
11	4	2	1.1	0.3	82	1.0	0.4	78
11	4	3	4.7	2.8	476	5.0	1.6	356
11	4	4	1.0	0.0	46	0.9	0.0	34
11	4	5	3.3	3.2	462	3.8	3.1	462
11	5	1	3.7	2.5	406	4.1	2.4	405
11	5	2	0.8	0.4	79	0.9	0.5	80
11	5	3	4.7	3.3	524	4.8	3.4	528
11	5	4	1.0	0.0	46	1.3	0.0	51
11	5	5	3.2	1.9	326	3.5	1.8	319
12	1	3	5.7	2.6	492	6.1	2.4	490
12	1	4	1.0	0.0	46	1.3	0.0	51
12	1	5	3.7	2.5	406	4.1	2.4	405
12	2	1	4.5	2.3	417	4.9	2.1	410

Pat. no.	Day	Meal [1]	Dietitian			VoiceDiab		
			CU	PFU	kcal	CU	PFU	kcal
12	2	2	1.1	0.3	82	1.0	0.4	78
12	2	3	5.1	2.8	488	4.8	2.0	476
12	2	4	1.6	0.0	46	1.3	0.0	51
12	2	5	4.4	2.2	402	4.8	2.0	393
12	3	1	4.2	2.5	429	4.4	2.5	427
12	3	2	1.1	0.3	82	1.0	0.4	78
12	3	3	5.5	2.0	423	5.2	1.4	341
12	3	4	1.6	0.0	85	2.1	0.0	85
12	3	5	4.4	2.5	437	4.9	2.4	436
12	4	1	4.3	2.1	395	4.7	2.0	389
12	4	2	0.8	0.5	91	0.9	0.6	91
12	4	3	5.9	2.8	522	5.9	2.7	511
12	4	4	1.0	0.0	46	1.3	0.0	51
12	4	5	4.8	2.6	455	5.3	2.4	454
12	5	1	4.3	2.1	395	4.7	2.0	389
12	5	2	1.1	0.3	82	1.0	0.4	78
13	1	3	5.7	2.6	492	6.1	2.4	490
13	1	4	1.0	0.0	46	1.3	0.0	51
13	1	5	3.7	2.5	406	4.1	2.4	405
13	2	1	3.4	2.2	368	3.8	2.1	367
13	2	3	5.1	2.8	488	5.8	2.8	513
13	2	4	1.0	0.0	46	1.3	0.0	51
13	2	5	3.4	1.9	332	4.3	1.9	364
13	3	1	3.2	2.2	351	3.4	2.1	344
13	3	2	1.1	0.3	82	1.0	0.4	78
13	3	3	5.3	1.9	412	5.0	1.3	330
13	3	4	1.0	0.0	46	0.9	0.0	34
13	3	5	3.3	2.2	359	3.8	2.1	358
13	4	1	3.3	2.0	330	3.6	1.8	323
13	4	2	0.8	0.5	91	0.9	0.6	91
13	4	3	5.5	2.0	428	5.5	2.0	417
13	4	4	1.0	0.0	46	1.3	0.0	51
13	4	5	3.7	2.5	406	4.1	2.4	405
13	5	1	3.2	1.9	326	3.5	1.8	319
14	1	3	4.5	2.1	489	4.6	2.2	403
14	1	4	1.0	0.0	46	1.3	0.0	51
14	1	5	3.4	2.3	378	3.6	2.2	365
14	2	1	3.3	1.9	326	3.5	1.8	319
14	2	2	1.1	0.2	82	1.0	0.0	78
14	2	3	5.4	2.6	484	3.8	2.3	383
14	2	4	1.0	0.0	46	1.3	0.0	51
14	2	5	3.3	2.2	360	3.8	2.1	358
14	3	1	3.2	1.9	326	3.5	1.8	319
14	3	2	0.8	0.5	91	0.8	0.6	92
14	3	3	5.1	2.3	438	6.0	1.9	420
14	3	4	1.0	0.0	46	1.3	0.0	51
14	3	5	3.7	2.5	406	4.1	2.4	405
14	4	1	3.5	2.3	380	3.8	2.1	358
14	4	2	1.1	0.3	82	1.0	0.4	78
14	4	3	4.9	1.8	388	4.8	1.2	317
14	4	4	1.0	0.0	46	1.3	0.0	51
14	4	5	3.2	2.2	351	3.4	2.1	344
15	1	3	5.0	4.9	684	4.7	3.8	568
15	1	4	1.0	0.0	46	1.3	0.0	51

Pat. no.	Day	Meal [1]	Dietitian			VoiceDiab		
			CU	PFU	kcal	CU	PFU	kcal
15	1	5	4.6	3.1	500	5.0	3.0	497
15	2	1	4.3	2.8	456	4.7	2.6	450
15	2	2	1.2	0.4	98	1.3	0.5	99
15	2	3	7.3	4.5	750	8.0	4.1	728
15	2	4	1.0	0.0	46	1.3	0.0	51
15	2	5	4.4	2.9	474	4.9	2.8	474
15	3	1	4.3	2.5	432	4.6	3.4	523
15	3	2	1.2	0.6	111	1.3	0.6	111
15	3	3	6.1	3.7	621	7.2	3.2	599
15	3	4	1.0	0.0	46	1.2	0.0	46
15	3	5	4.8	2.9	492	5.3	2.8	492
15	4	1	4.6	3.1	502	4.9	2.8	474
15	4	2	1.2	0.5	98	1.3	0.5	99
15	4	3	6.5	3.2	586	6.2	2.0	444
15	4	4	1.0	0.0	46	1.3	0.0	51
15	4	5	4.2	2.9	466	4.6	2.8	460
16	1	3	4.7	3.3	521	4.8	2.7	463
16	1	4	1.0	0.0	46	0.9	0.0	34
16	1	5	3.7	2.5	406	4.1	2.4	405
16	2	1	3.3	2.2	359	3.8	2.1	358
16	2	3	5.0	1.9	397	5.5	1.4	355
16	2	4	1.0	0.0	46	0.9	0.0	34
16	2	5	3.5	2.2	371	3.8	2.2	368
16	3	1	3.2	2.4	374	3.5	2.3	367
16	3	3	5.4	2.3	460	4.2	2.3	402
16	3	4	1.0	0.0	46	0.9	0.0	34
16	3	5	3.7	2.5	406	4.1	2.4	405
16	4	1	3.3	3.2	462	3.8	3.1	462
16	4	2	0.9	0.4	77	1.0	0.4	78
16	4	3	4.6	2.5	446	4.4	1.3	308
16	4	4	1.0	0.0	55	1.2	0.0	48
16	4	5	3.6	2.0	397	3.9	2.4	395
16	5	1	3.7	2.5	406	4.1	2.4	405
16	5	3	5.6	2.3	454	5.4	2.4	451
17	1	3	4.7	3.3	521	4.8	2.7	463
17	1	4	1.0	0.0	46	0.9	0.0	34
17	1	5	3.7	2.5	406	4.1	2.4	405
17	2	1	3.3	2.2	359	3.8	2.1	358
17	2	3	5.0	1.9	397	5.5	1.4	355
17	2	4	1.0	0.0	46	0.9	0.0	34
17	2	5	3.3	2.4	379	3.6	2.5	396
17	3	1	3.2	2.2	361	3.4	2.1	344
17	3	3	5.4	2.3	460	5.5	2.4	461
17	3	4	1.0	0.0	46	1.3	0.0	51
17	3	5	3.7	2.5	460	4.1	2.4	405
17	4	1	3.3	3.2	462	3.8	3.1	462
17	4	3	4.6	2.5	446	4.4	1.3	308
17	4	5	3.3	1.9	325	3.6	1.8	323
17	5	1	3.7	2.5	406	4.1	2.4	405
17	5	3	5.6	2.3	454	5.4	2.4	451
18	1	3	4.5	3.5	538	4.4	3.0	480
18	1	4	1.0	0.0	46	0.9	0.0	34
18	1	5	3.3	2.3	356	3.6	2.2	365
18	2	1	3.4	1.9	334	3.7	1.9	337

Pat. no.	Day	Meal [1]	Dietitian			VoiceDiab		
			CU	PFU	kcal	CU	PFU	kcal
18	2	3	5.0	3.1	515	5.4	1.7	379
18	2	4	1.0	0.0	46	1.3	0.0	51
18	2	5	3.2	2.2	351	3.4	2.1	344
18	3	1	3.2	2.6	396	3.5	2.5	389
18	3	2	0.8	0.2	57	0.8	0.5	83
18	3	3	5.3	2.7	486	6.5	2.4	501
18	3	4	1.0	0.0	46	1.3	0.0	51
18	3	5	3.4	1.9	334	3.9	1.8	333
18	4	1	3.4	1.9	334	3.9	1.8	333
18	4	2	0.9	0.4	77	1.0	0.4	78
18	4	3	4.8	2.0	395	5.1	1.5	353
18	4	4	1.0	0.0	46	1.3	0.0	51
18	4	5	3.2	2.2	351	3.4	2.1	344
19	1	3	6.0	2.9	529	6.3	2.8	529
19	1	4	2.0	0.4	131	2.0	0.3	112
19	1	5	5.3	3.3	534	5.5	3.2	534
19	2	1	5.3	3.3	534	5.5	3.2	534
19	2	3	5.0	3.3	534	5.3	2.3	447
19	2	4	2.0	0.4	131	2.0	0.3	131
19	2	5	5.5	3.1	539	5.9	3.1	545
19	3	1	5.4	3.2	539	5.9	3.0	539
19	3	2	1.0	0.7	114	1.2	0.7	114
19	3	3	7.5	4.4	746	7.7	3.9	701
19	3	4	2.0	0.4	131	2.0	0.3	112
19	3	5	5.9	3.6	605	6.5	3.4	605
19	4	1	5.4	3.2	539	5.9	3.0	533
19	4	2	1.0	0.4	98	1.3	0.5	99
19	4	3	5.8	5.1	750	5.3	4.0	603
19	4	4	2.0	0.4	131	2.0	0.3	112
19	4	5	5.4	4.4	661	6.1	4.2	661
19	5	1	5.9	3.2	568	6.5	3.1	568
19	5	2	1.0	0.2	69	1.1	0.3	78
19	5	3	7.5	2.4	551	6.8	1.6	429
20	1	3	5.8	3.2	563	6.0	3.2	556
20	1	4	1.0	0.0	46	0.9	0.0	34
20	1	5	3.2	1.9	326	3.4	1.9	323
20	2	1	3.2	1.9	326	3.5	1.8	319
20	2	3	5.2	3.1	524	5.2	3.1	522
20	2	4	1.0	0.0	46	1.3	0.0	51
20	2	5	3.3	2.2	359	3.8	2.1	358
20	3	1	4.7	2.5	452	5.2	2.4	446
20	3	3	7.6	2.3	540	7.3	2.2	536
20	3	4	1.0	0.0	46	1.3	0.0	51
20	3	5	4.0	3.2	486	4.4	3.2	493
20	4	1	4.2	1.9	372	4.6	1.8	360
20	4	3	6.5	2.4	510	5.9	2.7	506
20	4	4	1.0	0.0	46	1.3	0.0	51
20	4	5	4.2	3.3	503	4.7	3.2	510
20	5	1	4.2	1.9	372	4.6	1.8	360
20	5	2	3.1	1.9	324	3.2	1.1	238
21	1	3	5.8	3.2	563	6.0	3.2	556
21	1	4	1.0	0.0	46	0.9	0.0	34
21	1	5	3.2	1.9	326	3.5	1.8	319
21	2	1	3.2	1.9	326	3.5	1.8	319

Pat. no.	Day	Meal [1]	Dietitian			VoiceDiab		
			CU	PFU	kcal	CU	PFU	kcal
21	2	3	5.2	3.1	524	5.2	3.1	522
21	2	4	1.0	0.0	46	1.3	0.0	51
21	2	5	3.3	2.2	359	3.8	2.1	358
21	3	1	3.7	2.5	406	4.1	2.4	405
21	3	2	0.8	0.2	57	0.9	0.3	66
21	3	3	4.0	2.0	370	3.7	2.0	341
21	3	4	1.0	0.0	46	1.3	0.0	51
21	3	5	4.0	3.2	486	3.5	2.5	389
21	4	1	3.2	1.9	326	3.5	1.8	319
21	4	3	3.2	2.3	367	3.2	2.3	354
21	4	4	1.0	0.0	46	1.3	0.0	51
21	4	5	3.7	2.5	406	4.1	2.4	405
21	5	2	0.8	0.2	57	0.9	0.2	57
22	1	3	6.1	3.4	585	6.2	3.4	585
22	1	4	1.0	0.0	46	1.3	0.0	51
22	1	5	3.7	2.5	406	4.1	2.4	405
22	2	1	3.4	1.9	334	3.7	1.9	337
22	2	2	0.9	0.4	77	1.0	0.4	78
22	2	3	5.4	2.0	421	5.3	1.4	351
22	2	5	3.2	2.5	385	3.5	2.5	389
22	3	1	3.7	2.1	404	4.0	2.4	400
22	3	2	0.8	0.2	58	0.9	0.2	59
22	3	3	5.6	2.5	488	6.1	1.8	428
22	3	4	1.0	0.0	46	0.9	0.0	34
22	3	5	3.4	1.9	332	3.6	1.8	323
22	4	1	3.3	2.2	359	3.8	2.1	358
22	4	2	0.8	0.2	52	0.9	0.2	57
22	4	3	5.0	3.0	511	5.0	2.5	452
22	4	4	1.0	0.0	46	1.3	0.0	51
22	4	5	3.7	2.5	404	3.9	2.4	395
22	5	1	3.3	2.9	431	3.8	2.8	430
22	5	2	0.8	0.2	52	0.8	0.2	53
22	5	3	5.4	2.4	472	5.8	2.3	459
23	1	3	5.2	1.9	416	5.2	0.8	283
23	1	4	1.0	0.0	46	0.9	0.0	34
23	1	5	3.2	2.5	385	3.5	2.4	378
23	2	1	3.5	2.5	398	3.8	2.4	391
23	2	2	0.6	0.3	56	0.7	0.3	57
23	2	3	5.0	5.0	463	4.9	2.5	449
23	2	4	1.0	0.0	46	1.3	0.0	51
23	2	5	3.4	1.9	334	3.8	1.8	332
23	3	1	3.2	2.9	423	3.4	1.7	305
23	3	2	0.5	0.0	23	0.5	0.0	25
23	3	3	4.6	2.8	458	4.1	2.8	438
23	3	4	1.0	0.0	46	1.3	0.0	51
23	3	5	3.7	1.8	339	4.1	1.7	401
23	4	1	3.3	2.2	362	3.6	2.1	358
23	4	3	4.4	3.5	532	5.1	3.9	589
24	1	3	7.7	4.7	787	8.0	4.2	742
24	1	4	1.0	0.0	46	1.3	0.0	51
24	1	5	5.6	2.8	509	6.2	2.6	510
24	2	1	5.3	3.3	544	5.7	3.1	538
24	2	2	1.0	0.6	109	1.2	0.6	109
24	2	3	7.3	5.1	806	6.4	5.2	766

Pat. no.	Day	Meal [1]	Dietitian			VoiceDiab		
			CU	PFU	kcal	CU	PFU	kcal
24	2	5	5.8	3.0	541	6.2	3.0	547
24	3	1	5.4	2.8	502	5.6	2.8	502
24	3	2	0.9	0.0	84	1.0	0.4	85
24	3	3	6.3	4.1	669	5.9	2.9	525
24	3	5	5.4	3.3	552	5.8	3.2	558
24	4	1	5.8	3.0	541	6.4	2.8	541
24	4	2	1.0	0.0	92	1.1	0.5	92
24	4	3	5.9	3.3	573	6.1	3.3	574
25	1	3	5.2	1.8	399	5.3	1.5	362
25	1	4	1.0	0.0	46	1.3	0.0	51
25	1	5	3.2	1.9	326	3.5	1.8	319
25	2	1	3.3	2.3	373	3.5	2.3	366
25	2	3	4.9	2.1	416	4.6	2.1	389
25	2	4	1.0	0.0	46	1.3	0.0	51
25	2	5	3.3	2.2	359	3.8	2.1	358
25	3	1	3.2	1.9	326	3.9	1.8	333
25	3	2	0.8	0.6	91	0.9	0.6	97
25	3	3	5.9	3.0	540	4.3	2.4	412
25	3	5	3.7	2.5	406	4.3	2.4	412
25	4	1	3.4	1.9	334	3.9	1.8	333
25	4	2	0.6	0.3	56	0.7	0.3	57
25	4	3	5.0	2.6	463	4.8	2.0	391
26	1	2	1.7	0.4	110	1.9	0.3	109
26	1	3	5.0	2.3	438	5.1	2.7	471
26	1	4	1.0	0.0	46	1.3	0.0	51
26	1	5	3.5	1.9	341	4.0	1.8	340
26	2	1	3.4	2.2	360	3.7	2.1	356
26	2	3	4.7	2.3	448	4.8	1.5	347
26	2	4	1.0	0.0	46	1.3	0.0	51
26	2	5	3.5	2.2	367	3.9	2.1	365
26	3	1	3.3	2.2	357	3.6	2.1	353
26	3	3	6.0	2.8	531	5.9	2.1	443
26	3	4	1.0	0.0	46	1.3	0.0	51
26	3	5	3.7	2.5	404	4.0	2.4	400
26	4	1	3.4	1.9	334	3.9	1.8	333
26	4	2	0.9	0.0	77	1.0	0.4	78
26	4	3	5.4	1.9	512	5.4	2.4	452
26	4	4	1.0	0.0	46	1.3	0.0	51
26	4	5	3.4	1.9	332	3.7	1.8	328
27	1	3	4.5	2.5	435	5.4	1.5	354
27	2	4	1.0	0.0	46	1.3	0.0	51
27	2	5	3.5	2.3	380	3.9	2.2	379
27	3	1	2.2	0.7	166	2.5	0.7	166
27	3	2	0.9	0.4	77	0.9	0.4	77
27	3	3	3.2	2.4	382	2.9	1.8	293
27	3	4	1.0	0.0	46	1.3	0.0	51
27	3	5	2.3	1.5	247	2.7	1.4	246
27	4	1	2.2	0.7	166	2.4	0.6	159
27	4	3	4.7	2.8	477	4.7	2.9	475
27	4	4	1.0	0.0	46	1.3	0.0	51
27	4	5	2.6	1.8	293	2.9	1.8	292
27	5	1	2.4	0.7	174	2.5	0.6	172
28	1	3	5.2	1.9	408	5.3	1.9	408
28	1	4	1.0	0.0	46	1.3	0.0	51

Pat. no.	Day	Meal [1]	Dietitian			VoiceDiab		
			CU	PFU	kcal	CU	PFU	kcal
28	1	5	3.3	1.9	330	3.6	1.9	330
28	2	1	3.2	1.9	326	3.5	1.8	319
28	2	2	0.9	0.4	77	0.9	0.4	77
28	2	3	4.7	2.5	442	4.8	1.9	384
28	2	4	1.0	0.0	46	1.3	0.0	51
28	2	5	3.4	2.7	413	3.8	2.6	412
28	3	1	3.2	2.2	351	3.4	2.1	344
28	3	2	0.5	0.6	85	0.6	0.6	84
28	3	3	4.3	3.0	478	5.4	1.7	385
28	3	4	1.0	0.0	46	1.3	0.0	51
28	3	5	3.6	2.5	398	4.0	2.4	398
28	4	1	3.3	1.9	326	3.6	1.9	330
28	4	2	0.6	0.2	62	0.6	0.4	62
28	4	3	5.0	2.1	433	5.2	1.5	346
28	4	4	1.0	0.0	46	1.3	0.0	51
28	4	5	3.2	1.9	326	3.5	1.8	319
29	1	3	4.0	2.1	393	4.7	2.1	393
29	1	4	1.0	0.0	46	1.3	0.0	51
29	1	5	3.3	1.9	330	3.6	1.9	330
29	2	1	4.1	2.5	425	4.4	2.4	412
29	2	2	0.8	0.6	96	0.9	0.6	97
29	2	3	5.9	2.1	451	5.2	1.4	341
29	2	4	1.0	0.0	46	1.3	0.0	51
29	2	5	4.5	2.0	392	4.4	1.9	386
29	3	1	3.7	1.5	311	3.8	1.2	314
29	3	2	1.6	0.2	108	1.5	0.3	106
29	3	3	5.9	2.3	472	5.9	2.1	447
29	3	4	1.0	0.0	46	0.9	0.0	34
29	3	5	4.5	2.0	392	4.7	2.1	390
29	4	1	3.6	2.5	408	3.7	2.4	388
29	4	2	0.8	0.3	67	0.9	0.3	68
29	4	3	5.3	2.2	437	5.9	2.1	447
30	1	1	4.2	1.9	365	4.7	1.8	363
30	1	2	0.9	0.4	77	1.0	0.4	82
30	1	3	5.2	3.2	528	5.3	3.2	529
30	1	4	1.0	0.0	46	1.3	0.0	51
30	1	5	3.2	1.9	326	3.5	1.8	319
30	2	1	4.9	2.6	465	5.5	2.4	461
30	2	2	0.8	0.3	67	0.9	0.3	68
30	2	3	5.9	2.8	520	6.6	1.8	435
30	2	4	1.0	0.0	46	1.1	0.0	44
30	2	5	3.6	2.5	400	4.0	2.5	416
30	3	1	4.4	1.9	378	4.9	1.8	374
30	3	2	0.9	0.4	77	1.0	0.4	78
30	3	3	5.2	2.9	510	5.5	1.6	381
30	3	4	1.0	0.0	46	1.3	0.0	51
30	3	5	3.4	2.4	387	3.8	2.4	406
30	4	1	4.3	2.9	477	5.0	2.8	476
30	4	2	0.8	0.4	80	0.9	0.5	80
30	4	3	5.6	2.4	400	5.7	1.7	393
30	4	4	1.0	0.0	46	1.3	0.0	51
30	4	5	3.2	1.9	326	3.5	1.8	319

[1] Meal codes: 1 – breakfast, 2 – morning snack, 3 – lunch, 4 – afternoon snack, 5 – dinner.
